# Advances and challenges in leukemia treatment: A focus on monoclonal antibodies and emerging therapies

**DOI:** 10.32604/or.2025.055100

**Published:** 2025-05-29

**Authors:** GIOVANA GOMES CHAGAS, RUAN PIMENTA, NAYARA IZABEL VIANA

**Affiliations:** 1 Department of Bioscience, Universidade do Estado de Minas Gerais-UEMG, Passos, MG 31630, Brazil; 2 Precision Immunology Institute, Department of Immunology and Immunotherapy, and Tisch Cancer Institute, Icahn School of Medicine at Mount Sinai, New York, NY 10029, USA

**Keywords:** Antibodies, Chronic or acute lymphoid leukemia, Immunoglobulins, Immunotherapy, Leukemia treatment and monoclonal antibodies

## Abstract

The monoclonal antibodies consist of an innovative form of immunotherapy, capable of defeating several diseases, such as cancer. It is an emergent and important theme, that advances evaluation, challenges, and future perspectives with high relevance to identify gaps in recent studies and to consolidate this general theme in only one research. Its action in Chronic and Acute Lymphoid Leukemia has been evaluated in several clinical trials, which were selected between 2022 and 2023, in order to understand better the monoclonal antibodies that were most studied. The biopharmaceutical compounds Ibrutinib, Obinutuzumab, Rituximab, Venetoclax, and Inotuzumab Ozogamicin were the ones that most appeared in the most recent publications, indicating the importance of amplifying the studies. The action mechanisms that are used imply that their combined use has more success in the disease remission, showing a lower recurrence, adverse effects, and toxicity. Besides the adverse effects and overwhelming prices of the treatment, these immunotherapies results are promising, amplifying the survival rates, improving the patient’s life quality, and resulting in a precision medicine, aiming a custom treatment. The future perspectives on this therapy consist of its application in the public health system, with patients being able to be submitted to this treatment without any costs and receive a better life quality.

## Introduction

Cancer remains a major focus in medicine due to its complexity and lack of cure, affecting nearly every area of the human body with over 277 types and subtypes arising from genetic mutations that alter cell functions [1]. Its treatment is challenging and varies significantly depending on the cancer type and stage at diagnosis [2].

Leukemia, in particular, is notable as it affects hematopoietic and lymphoid tissues through uncontrolled proliferation of hematopoietic stem cells in the bone marrow, multiplying and surviving more than other cells. This type of cancer can dangerously affect the patient’s health, able to evolve into a death scenario. Among many types of lymphoid malignancies, acute lymphoblastic leukemia, chronic lymphocytic leukemia, and non-Hodgkin lymphoma are most relevant to this review [2,3].

Acute Lymphoblastic Leukemia (ALL) arises from a genetic error in hematopoietic stem and progenitor cells giving rise to leukemia blast cells, which are able to proliferate continuously, suppressing healthy cell development by the lack of division. ALL is prevalent in both adults and children, presenting symptoms like fever, lethargy, bleeding, and lymphadenopathy [4]. Chronic Lymphocytic Leukemia (CLL) is an acquired and not hereditary disease, often discovered incidentally during routine examinations, especially in adults, due to a lack of symptoms. In this case, the healthy cells are not suppressed, while the malignant B lymphocytes keep their disordered proliferation [5].

Leukemia treatments typically involve chemotherapy, a procedure that can harm both cancerous and healthy cells. Consequently, recent research is continuing to explore immunotherapy using Monoclonal Antibodies (mAbs), which can target tumor cells while sparing healthy ones [6].

mAbs are produced by identical B lymphocyte clones, each specific to a single antigen epitope, enhancing their ability to target and eliminate tumor cells. This specificity minimizes side effects compared to traditional chemotherapy and improves patient quality of life [6,7] mAbs are administered intravenously or orally and work through the immune system to combat cancer effectively. mAbs represent a promising alternative in cancer treatment, with ongoing studies aimed at optimizing their therapeutic efficacy across various cancer types.

mAbs are highly effective biopharmaceuticals for leukemia treatment, offering specificity that can reduce the aggressiveness and side effects compared to chemotherapy or radiotherapy. Despite their promising advances, significant challenges remain, requiring ongoing research. This review consolidates recent studies on mAbs, detailing their mechanisms of action, potential adverse effects, and limitations, to provide a comprehensive overview of current knowledge, identify research gaps, and guide future research and education for healthcare professionals.

The primary objective of this comprehensive literature review is to explore the role and potential of laboratory-produced and already commercially used mAbs in the treatment of two types of leukemia: CLL and ALL. This review aims to achieve the following specific objectives: Firstly, it will provide a descriptive analysis of the different types of mAbs, and drugs used in the treatment of CLL and ALL, highlighting their mechanisms of action and clinical efficacy. Secondly, it will investigate recent advances in the laboratory production of mAbs, focusing on the latest techniques to enhance their therapeutic potential. Thirdly, the review will evaluate the challenges and limitations associated with the application of mAbs in leukemia treatment, such as cost considerations, production complexities, and potential adverse effects. Additionally, it will discuss the potential benefits and challenges of combining mAbs with other therapeutic agents, such as chemotherapy drugs, small molecule inhibitors, and immunomodulatory treatments, to enhance efficacy and overcome resistance. Finally, the review aims to identify research gaps and suggest future directions for developing more effective and safer mAbs for leukemia treatment, including innovative approaches to improve specificity, reduce side effects, and enhance patient outcomes. By addressing these objectives, the review aims to provide a comprehensive overview of the current state and future prospects of mAbs in the treatment of CLL and ALL, contributing valuable insights for ongoing and future research in this field.

## Literature Review

After reviewing the articles, it was observed a frequent appearance of three Abs and two inhibitor molecules allowed us to understand which of these biopharmaceuticals, including monoclonal antibodies and other types, have been more studied and are gaining more prominence, which are: Ibrutinib, Inotuzumab Ozogamicin, Obinutuzumab, Rituximab, and Venetoclax.

The aforementioned three mAbs, used alone or in combination with other drugs have shown satisfactory performance in terms of remission of Chronic Lymphocytic Leukemia and Acute Lymphoblastic Leukemia, implying few side effects. Each one presents different mechanisms of action (Table 1), functional combinations, and associated symptoms.

**Table 1:** Description of the action mechanism, targets, and the possible and ideal combinations of the mAbs and drugs selected for the integrative review

Drugs	Monoclonal antibodies	Action mechanism	Targets	Possible combinations	Ideal combinations
Ibrutinib	**-**	Bruton tyrosine kinase (BTK) inhibitor, reduces B cells, consequently reducing tumors.	BTK	Obinutuzumab	Obinutuzumab
**-**	Obinutuzumab	It binds to the CD20 antigen, inducing cell death and antibody-dependent cellular phagocytosis, destroying malignant B Lymphocytes in various ways.	CD20	Ibrutinib, Clorambucil, Venetoclax and Entospletinib	Ibrutinib and Venetoclax
**-**	Rituximab	It binds to the CD20 antigen, inducing cell death and antibody-dependent cellular phagocytosis, destroying malignant B Lymphocytes in various ways.	CD20	Venetoclax	Venetoclax
Venetoclax	**-**	Inhibits the expression of B-cell lymphoma protein 2 (BCL-2), causing programmed cell death of B cells.	BCL-2	Obinutuzumab, Ibrutinib and Rituximab	Obinutuzumab and Ibrutinib
**-**	Inotuzumab Ozogamicin	It binds to CD22, enabling the activation of N-acetyl-gamma-calicheamicin dimethylhydrazide, breaking the double strand of DNA, shutting down cellular functions, and inducing apoptosis.	CD22	Blinatumomab	Blinatumomab

The comparative analysis of the findings indicates that the combination of different mAbs produces distinct effects across individual patients. In other words, depending on the combination performed, adversities may be more or less intense, and toxicity may be reduced or increased. The use of these combined biopharmaceuticals is mostly positive, as it is a recent strategy in oncological therapies that may represent a significant advancement in the studies being conducted.

Each type of antibody targets specific mechanisms associated with different leukemias. In Chronic Lymphocytic Leukemia (CLL), antibodies and therapeutic drugs focus on targets such as BTK, CD20, and BCL-2. In Acute Lymphoblastic Leukemia (ALL), relevant targets include CD20 and CD22.

Each mAb (monoclonal antibody) or medicine functions according to its specific mechanism of action, which can cause cell death in different ways, including inducing apoptosis, which function through two main pathways: extrinsic and intrinsic. The extrinsic pathway operates via surface receptors, while the intrinsic pathway involves mitochondria, as seen with Venetoclax. Thus, it utilizes distinct cellular components and engages different cells of the immune system to effectively promote apoptosis.

Ibrutinib is a molecule that inhibits Bruton’s Tyrosine Kinase (BTK). BTK plays a crucial role in the development of B cells by regulating their differentiation into mature B cells, which are essential for producing antibodies. Specifically, BTK is involved in the signaling pathways that guide this maturation process. In the context of leukemia, dysregulation or overactivity of BTK can contribute to the expansion of malignant B cells. Therefore, by inhibiting BTK, Ibrutinib reduces the proliferation of these aberrant B cells, thereby helping to manage leukemia consequently, reducing tumors related to CLL.

The combination of Ibrutinib and Obinutuzumab for the treatment of leukemia, used for a total of 15 months, resulted in high survival rates and low long-term toxicity, unlike the continuous use of Ibrutinib alone, which can cause severe toxicity as well as cardiac disorders and bleeding [8].

Obinutuzumab is a mAb that acts as an immunomodulator. Its mechanism of action is related to the CD20 antigen, which is expressed in B lymphocytes. Its action is based on binding to this antigen, inducing cell death, antibody-dependent cytotoxicity or complement-dependent cytotoxicity, and antibody-dependent cellular phagocytosis, thus destroying malignant B lymphocytes in various ways.

This referenced biopharmaceutical can be combined with Chlorambucil. Clinical studies comparing the combination of Obinutuzumab with Ibrutinib and Obinutuzumab with Chlorambucil have shown that the combination with Ibrutinib results in greater efficacy, higher disease remission rates, fewer adverse effects, and lower rates of tumor recurrence. Specifically, the combination of Obinutuzumab with Ibrutinib has been found to be more effective in achieving remission and maintaining long-term disease control compared to the combination with Chlorambucil. Additionally, this combination has demonstrated fewer side effects, making it a preferable choice for treating elderly patients, with or without mutations, especially those with high-risk genomic characteristics [9]. Comparing these combinations is crucial to identifying the optimal treatment strategy for patients affected by the disease. Combination therapy with Obinutuzumab and Ibrutinib not only improves clinical outcomes but also enhances patient safety and quality of life.

An assessment of triple combination was also possible, using Ibrutinib and Obinutuzumab combined with Venetoclax. Since the initial combination has already shown benefits, the addition of Venetoclax suggests a potential therapy for patients with del (17p) and/or TP53 mutations, as well as for those who are currently untreated.

Obinutuzumab can also be used in combination with Entospletinib, which is a SYK inhibitor, the Spleen Tyrosine Kinase, preventing the activation of leukemic B cells. Clinical trials highlight the importance of this combination, as it can overcome resistance to Ibrutinib and Venetoclax and, the adverse effects associated with them [10]. In addition, this combination is also able to stop T cell immunosuppression, reducing the burden of Chronic Lymphocytic Leukemia [11].

Venetoclax also functions as an inhibitor, targeting the BCL-2 protein, which aids in the survival of B cells due to its anti-apoptotic ability [12], and also shows resistance against other cancer therapies, such as chemotherapy. Thus, by inhibiting the expression of this protein, Venetoclax carries out programmed cell death of B cells.

Its combination with Ibrutinib and Obinutuzumab was positive, and thus, other clinical trials focused on evaluating the effectiveness of the combination of Venetoclax and Obinutuzumab alone, whose treatment is currently allowed [13] Studies aimed to assess the optimal dosage and timing for better treatment, and Venetoclax administered at 400 mg combined with 1000 mg of Obinutuzumab resulted in a safety profile with efficacy [14].

Rituximab is yet another therapy option for Chronic Lymphoid Leukemia and Acute Lymphoid Leukemia, which when combined with Venetoclax itself, is ideal for young patients and/or those with unfavorable genetic alterations. This treatment is easily administered, with high tolerance and positive treatment responses with low recurrence rates. Rituximab has a mechanism of action similar to Obinutuzumab, binding to the CD20 antigen, causing cell death mediated by complement-dependent cytotoxicity and antibody-dependent, cell-mediated cytotoxicity, as well as apoptosis. However, the difference between both mechanisms of action is the way in which the binding to the antigen occurs, activating different B cell receptor pathways.

This biopharmaceutical can also be used in cancer treatment, either alone or in combination with other therapies, such as chemotherapy. Its effectiveness as a monotherapy has been tested according to standard clinical protocols, with 4 doses administered over the course of treatment. However, its performance was not effective, likely requiring prolonged administration [15]. ALL persisted, with no significant survival rate. This factor, as demonstrated by recent studies, highlights the importance of combining antibodies with chemotherapy in cancer therapies. Evidence shows that combination therapies tend to achieve better outcomes compared to monotherapies by targeting multiple pathways simultaneously and overcoming potential resistance mechanisms [16]. However, when Rituximab was tested using a dose-escalation methodology, in combination with chemotherapy and a different timeframe, it yielded satisfactory results, potentially enhancing clinical outcomes. ALL showed remission in approximately 97% of patients undergoing treatment, while 2 patients died from treatment complications [17]. Thus, the need for further studies involving Rituximab associated with chemotherapy becomes visible since, depending on the methodology used, it can be beneficial to the patient.

Given the advantage of combining antibodies, one study used the combination of Rituximab and Acalabrutinib to test patients who had not yet started treatment for Chronic Lymphocytic Leukemia. The research was conducted during the pandemic period, which allowed the treatment to be administered at home without the need for travel. The dose infusion was slow and in small quantities, and in a few patients, there was an adverse reaction, which, however, did not preclude continued treatment. Moreover, the study demonstrated that this medicine can indeed be self-administered safely and effectively, allowing for the perception of an applicability parameter for the treatments studied [18].

Inotuzumab Ozogamicin is an anti-CD22 antibody (Inotuzumab) conjugated to the cytotoxic drug Ozogamicin, used in the treatment of certain hematologic malignancies. It was first evaluated in B-cell lymphomas but was subsequently shown to be highly effective in ALL. Known for its ability to target CD22, a protein expressed on the surface of B-cells, including malignant B-cells, it has proven to be an effective therapy in these conditions. This drug combines the targeting capabilities of a monoclonal antibody with the cytotoxic effects of a potent chemotherapeutic agent. Another mAbs mostly acts solo, being well-tolerated and presenting a low incidence of infections caused by the treatment itself. Inotuzumab Ozogamicin has a mechanism of action by binding to CD22, which is expressed by the tumor, the B Cells. This binding allows for the activation of N-acetyl-galactosamine dimethylhydrazide, which breaks the double-stranded DNA, terminating cellular functions and inducing apoptosis [19–21], as can be observed in the referenced studies.

Studies indicate that Inotuzumab is a good medicine for disease remission, which, after treatment completion, can still delay or prevent a relapse [22]. Its use is recommended at the beginning of ALL treatments. Despite having benefits, the treatment can be more beneficial and present fewer adverse factors when combined with other mAbs treatments, such as mini-hyper-CVD chemotherapy and Blinatumomab [23].

Testing Inotuzumab Ozogamicin alone with low-intensity mini-hyper-CVD chemotherapy yielded satisfactory results in ALL, preventing disease relapse. However, when combined with Blinatumomab, the survival rate increases, further benefiting treatment outcomes [19–21]. This is another indication of the importance of combining mAbs in ALL treatments.

This variation in results and therapies among different monoclonal antibodies (mAbs) in the treatment of leukemia highlights the significance of personalized medicine [24]. By tailoring treatments to individual patients, we advance toward precision medicine, which aims to achieve the highest possible remission rates, minimize relapses, and reduce adverse effects [25].

Besides its advantages, this type of therapy presents some limitations, like the adverse effects or the prices attributed to each biopharmaceutical [26]. The website Medicine Consultation presents the prices in Reais (Brazilian currency), and functions gathering information from many suppliers and selling the products. This content is shown in Table 2, where the prices observed are impractical, overcoming the Brazilian minimum wage many times, compromising the treatment viability, and not being able to be used either in public or private health centers.

**Table 2:** Prices associated to each mAb or medicine

Monoclonal antibodies	Drugs	Commercial name	Dose	Price
–	Ibrutinib	Imbruvica	140 mg, 30 pills	R$ 17,250.00
Obinutuzumab	**-**	Gazyva	40 mL	R$ 32,250.00
Inotuzumab Ozogamicin	**-**	Besponsa	1 mg	R$ 87,670.27
–	Venetoclax	Venclexta	50 mg, 7 pills	R$ 1636.90
Rituximab	**-**	Vivaxxia	50 mL	R$ 5100.00

Currently, several mAbs have been already approved for use and undergoing clinical trials in the country, but patients face those limitations, before or during their treatment. In addition, ANVISA (Brazil’s regulatory agency) permitted the use of CAR-T cells as well for leukemia treatment in 2022. This involves genetically altering the patient’s T lymphocytes and modifying and reprogramming them to destroy tumors.

## Conclusion

Treating leukemia with mAbs offers a promising alternative, showing positive trends in disease remission with generally milder adverse effects compared to traditional chemotherapy. Combining two or more mAbs and drugs has proven beneficial in reducing symptoms and toxicity, achieving remission by neutralizing harmful factors during treatment.

Studies from 2022 to 2023 highlighted mAbs like Obinutuzumab, Rituximab, and Inotuzumab Ozogamicin, and drugs such as Venetoclax and Ibrutinib. These biopharmaceuticals target different proteins to induce apoptosis, complementing each other and enhancing their overall effectiveness. Ongoing research in this area shows promise, in improving patient’s quality of life and survival rates. Further studies are needed and recommended to explore new combinations and antibodies, aiming for more effective treatments with fewer long-term toxic effects.

Personalized therapy, tailored to the patient’s genetic and immunological profiles, offers a more effective approach. Despite challenges such as drug pricing, adverse effects, and limited laboratory resources, future perspectives on mAbs present valuable tools for advancing cancer treatment, aiming for less toxic and more effective therapies, as well, as being applied in the public health system, with patients being able to be submitted to this treatment without any costs, and acquiring more life quality.

## Data Availability

Data sharing not applicable to this article as no datasets were generated or analyzed during the current study.

## Abbreviation

BCL-2: B-cell lymphoma protein 2
CLL: Chronic Lymphocytic Leukemia
ALL: Acute Lymphoblastic Leukemia
mAbs: Monoclonal Antibodies
BTK: Burton’s Tyrosine Kinase
